# Next generation sequencing-aided comprehensive geographic coverage sheds light on the status of rare and extinct populations of *Aporia* butterflies (Lepidoptera: Pieridae)

**DOI:** 10.1038/s41598-020-70957-4

**Published:** 2020-08-18

**Authors:** Valentina Todisco, Raluca Vodă, Sean W. J. Prosser, Vazrick Nazari

**Affiliations:** 1 Unaffiliated, Marino (Rome), Italy; 2 Unaffiliated, Torino, Italy; 3 Unaffiliated, Guelph, ON N1E 7L4 Canada; 4 Unaffiliated, Ottawa, ON K2M 2Y1 Canada

**Keywords:** Ecology, Evolution, Molecular biology, Zoology

## Abstract

The Black-veined White *Aporia crataegi* (Linnaeus, 1758), a common and widespread butterfly ranging from northwestern Africa to Europe and Asia, has been extinct in Britain since the 1920s and is on a steady decline in several other parts of its range. In order to investigate genetic diversity within *A. crataegi* and its correspondence with current subspecies-level taxonomy, we barcoded 173 specimens from across its range including, for the first time, extinct populations from Britain and Korea. Using next generation sequencing we also obtained a sequence for *Aporia joubini*, a peculiar taxon from China known only by its type specimen collected in the early twentieth century. Our phylogenetic analysis placed *A. joubini* sister to *A. oberthuri*, although further taxon sampling may reveal a different scheme. Within *A. crataegi*, we observed a shallow and weak mitogenomic structure with only a few distinct lineages in North Africa, Sicily, Iran, and Japan. Eurasian populations, including those extinct in Britain and Korea, clustered into a large set of closely allied lineages, consistent with a recent expansion during the Late Pleistocene glacial period. This study highlights the importance of museum collections and the unique opportunities they provide in documenting species diversity and helping conservation efforts.

## Introduction

Human activities threaten considerable numbers of species and natural populations that, in turn, significantly affect ecosystem functioning^1,2^. Each species contains immense amounts of genetic information within its geographical populations, and this intra-specific genetic diversity not only is a repository of past geological, climatic, and environmental information, but also influences population persistence and evolutionary potential^3^. Thus, the resulting permanent loss of genetic information due to local extinctions not only affects evolutionary potential under changing environmental conditions, but also impedes a full understanding of the evolutionary history of species. Natural history collections have long been used by morphologists and taxonomists to probe the evolutionary process and describe biological diversity. These biological archives also offer great opportunities for genetic research in taxonomy, conservation, systematics, and population biology and they represent an invaluable resource for studying extinct taxa.

Next Generation Sequencing (NGS) has revolutionized nearly every field of genetics, but few have profited from it as much as ancient DNA research. NGS has increased the amount of DNA sequence data available from extinct organisms by several orders of magnitude and is extensively used in phylogenetic studies using museum specimens [e.g.^4,5^].

The Black-veined White, *Aporia crataegi*, is a well-known butterfly widespread in orchards and bushy places in the Palearctic region, from north-western Africa to Japan, across Europe and Asia, although very localized and uncommon in some areas^6–8^. Its larvae feed on Rosaceae such as *Prunus, Pyrus*, and especially *Crataegus*. Despite a very simple and uniform wing pattern, numerous local subspecies have been described under *A. crataegi* across its range*,* and the status of many remain doubtful: Della Bruna et al.^9^ listed 25 subspecies under *A. crataegi*, while Savela^10^ recognized 23. The Black-veined White belongs to a diverse genus composed of numerous other species, most of which are confined to southwest China and the Himalaya-Hengduan mountain regions. The phylogenetic position of *Aporia* within Pieridae has been well established^11,12^. However, its monophyly has been recently questioned by Ding and Zhang^13^, who found that the monotypic genus *Mesapia* Gray, 1856 (type species: *Pieris peloria* Hewitson, [1853]), proposed either as a subgenus of *Aporia*^14,15^ or as a distinct genus^9,16,17^, is nested within *Aporia* sensu lato (see also^18^). This may highlight a need to synonymize *Mesapia* under *Aporia*. Moreover, Ding and Zhang^13,18^ found that another subgenus within *Aporia*, subgn. *Metaporia* (Butler 1870, type species: *Pieris agathon* Gray, 1831) which includes species with a bifid apex of the uncus in male genitalia sensu Della Bruna et al.^19^, is also paraphyletic, and therefore subdivisions of *Aporia* into separate subgenera cannot be supported. Two studies have so far investigated the phylogeny and evolution of the species within this genus, using either a mitochondrial COI fragment alone^20^ or accompanied by a second nuclear gene^18^, although taxon sampling in both of these studies was incomplete. *Aporia crataegi* was once a fairly widespread and locally plentiful butterfly in southern England and Wales. By 1872 it had disappeared from Kent, and according to Allan^21^, the last authentic British record as a native species was a single specimen caught in New Forest in 1880, although it is more widely accepted that the last British specimens were those from Herne Bay in Kent caught during the 1920s. The reasons behind the extinction of *A. crataegi* in Britain remain unknown, and attempts to reintroduce it by releasing hundreds of continental specimens in Kent and elsewhere in Britain failed to re-establish *A. crataegi* on a permanent basis. The populations of *A. crataegi* can fluctuate greatly in numbers over time, but the reasons for this are not fully understood. Major endangerment factors for their decline include habitat destruction, climatic change, parasitism, bird predation, and the use of pesticides (especially herbicides) in orchards^22,23^. In recent years, strong declines in distribution, or population size of more than 30%, have been recorded for *A*. *crataegi* from Austria, Romania, Ukraine, Albania, France, Latvia, Norway, Serbia, Algeria, and Morocco^24^, and the butterfly is also reported extinct from Netherlands, Luxembourg, Czech Republic^25^, and Korea (since 1997:^23^). In this study we analysed the mitochondrial COI barcode region for 23 out of 25 subspecies of *A. crataegi* as recognized by Della Bruna et al.^9^ from the Palearctic region, including, for the first time, the extinct British populations. We also obtained a COI barcode from the only known specimen of *A. joubini,* collected in 1902 from China and now hosted in the Natural History Museum of London (NHMUK type). We aimed to: (i) investigate the phylogenetic position of *A. joubini* within the *Aporia* genus, (ii) assess the phylogeographic patterns within *A. crataegi*, (iii) investigate if the current taxonomic entities correspond with these patterns, and (iv) evaluate a continental vs. an autochthon origin of the British specimens.

## Materials and methods

### Taxon sampling

We examined 173 *A. crataegi* individuals representing 23 subspecies out of the 25 recognized by Della Bruna et al.^9^ from sites across Eurasia and North Africa (Maghreb region) (Fig. 2A and Supplementary Dataset File). We were unable to obtain sequences from ssps. *fert* (Rhodes Island, Greece) and *sachalinensis* (Sakhalin, Russia). We analysed ten British individuals: four collected between 1860 and 1870, before the extinction of the native populations, and six at the beginning of the twentieth century, supposedly re-introduced from the continent. In addition, we analysed four specimens from Iranian populations of *A. leucodice,* and the only known (type) specimen of *A. joubini*, collected in 1902 from China.

### DNA extraction

A single leg was removed from each specimen and sent to the Centre for Biodiversity Genomics in Guelph, Canada for DNA extraction, amplification, and sequencing. DNA was extracted from a single leg on a Biomek FX liquid handling robot using a semi-automated DNA extraction protocol^26^ with glass fiber plates (PALL Acroprep 96 with 3 µm GF membrane over 0.2 µm Bioinert membrane). DNA was eluted in 30–40 µl of ddH20 pre-warmed to 56 °C and stored at − 80 °C.

### Next‐generation and Sanger sequencing

Due to DNA degradation in the very old specimens of *A. joubini* and the British *A. crataegi*, the 658 bp COI barcode region was recovered using an NGS-based protocol^27^. In brief, for each sample, multiple short, overlapping amplicons were generated using nested, multiplex PCR. In order to associate reads with their source specimen, the amplicons were tailed with sample-specific universal molecular identifiers (UMI) before being pooled for sequencing on an Ion Torrent PGM. The short sequence reads were attributed to a sample via the UMIs, and filtered for quality and length before being assembled into a single barcode sequence.

For the remaining (younger) specimens, PCR and Sanger sequencing were carried out following standard procedures for Lepidoptera^28,29^. Briefly, the 658 bp barcode region was amplified using the primers LepF1 (5′ ATTCAACCAATCATAAAGATATTGG 3′) and LepR1 (5′ TAAACTTCTGGATGTCCAAAAAATCA 3′) and sequenced on an ABI 3730XL (Applied Biosystems capillary sequencer). The trace files were edited using CodonCode Aligner 6.0.2 (CodonCode Corporation, Dedham, Massachusetts) and all resulting mtDNA sequences were aligned using the same program. All sequences were submitted to GenBank (Accession Numbers in Supplementary Dataset File) and BOLD system repository (dataset name: DS—APORIA; https://doi.org/10.5883/DS-APORIA).

### Data set compilation and tree reconstruction procedures

To assess the phylogeographic patterns within *A. crataegi* and the origin of the British specimens, we complemented the 173 specimens sequenced specifically for this study with an additional 24 sequences of *A. crataegi* from public DNA barcoding projects (see Supplementary Dataset File). The phylogenetic position of *A. joubini* within the genus *Aporia* was further assessed, reconstructing the phylogenetic relationships in the subtribe Aporiina. Seventeen GenBank sequences (n = 17) from 12 species of *Aporia* and 6 outgroups, mainly selected from Ding and Zhang^18^, were thus added (see Supplementary Dataset File). We excluded *A. martineti* (KU921265), *A. genestieri* (KU921259) and *Mesapia peloria* (KU921272) sequences from Ding and Zhang^18^ due to the questionable quality of the COI sequences in GenBank. All sequences were aligned using CodonCode Aligner 6.0.2. Haplotype and nucleotide diversity were calculated using DnaSP 5.0^30^. The mean p-distance between groups and its variance (bootstrap method, 500 replicates) was calculated using MEGA 10^31^. To represent the genealogical relationships among mtDNA haplotypes, we calculated a Median Joining (MJ) network using NETWORK 5^32^. *Aporia* relationships were assessed via Maximum Likelihood (ML). The software package ModelFinder^33^ in IQ-TREE 1.6.11^34^ was used to select the best fitting model of evolution by Bayesian information criterion scores (BIC). The ML analysis was carried out with RaxML 7.0.3^35^, on the T-REX (Tree and reticulogram REConstruction) web server (^36^; https://www.trex.uqam.ca), with the rapid hill-climbing algorithm and 1,000 non-parametric bootstrap inferences. The highest bootstrap values were obtained setting the model of evolution to GTRCAT, searching for the best-scoring ML tree in a single run. The best ML tree was visualized and edited with FigTree version 1.4^37^.

### Tests of demographic equilibrium and population expansion in mtDNA haplogroup

To test demographic equilibrium or events of recent expansion in *A. crataegi*, different sets of mtDNA sequences were selected (Table 1) considering the results of the previous phylogenetic analysis. To detect departures of DNA sequence variability from the expectations of the neutral theory of evolution^38^, R2^39^ and Fu’s FS^40^ parameters were calculated. Both have been shown to provide sensitive signals of historical population expansions^40^. While the latter is based on the haplotype distribution and it is expected to be strongly affected by recombination, R2 is based on mutations and likely to be conservative on recombining regions^41^. Moreover, Fu’s simulations suggest that FS is a more sensitive indicator of population expansion than other parameters, therefore FS should be regarded as significant if P < 0.02^42^. Arlequin 3.5^43^ and DnaSP 5.0^30^ were used to compute FS (P < 0.02) and R2 (P < 0.05) respectively, and to test their statistical significance by simulating random samples (10,000 replicates) under the null hypothesis of selective neutrality and constant population size using coalescent algorithms (both modified from Hudson^44^). Expected mismatched distributions and parameters of sudden expansion τ = 2 μ t (τ, sudden demographic expansion parameter; t, true time since population expansion; μ is the substitution rate per gene) were calculated using Arlequin 3.5 by a generalized least-squares approach^45^, under models of pure demographic expansion and spatial expansion^46,47^. The probability of the data under the given model was assessed by the goodness-of-fit test implemented in Arlequin 3.5. Parameter confidence limits were calculated in Arlequin 3.5 through a parametric bootstrap (1,000 simulated random samples).

**Table 1:** Results of the tests of demographic equilibrium and mismatch analysis in mtDNA phylogeographic groups.

Group	*N*	*H*	*h*	*Fs*	P	*R*_2_	P	τ	τ (5%)	τ (95%)	t (ka)	t (5%)	t (95%)
Eurasian haplogroup	114	24	0.65 ± 0.04	**− 29.24**	**0**	**0.095**	**0**	**1.011**	**0.746**	**1.492**	**67**	**49**	**99**
Mediterranean haplogroup	35	12	0.75 ± 0.07	**− 6.99**	**0**	**0.12**	**0**	**1.748**	**0.869**	**2.895**	**116**	**57**	**191**

*N*, number of mtDNA sequences; *H*, number of unique mtDNA haplotypes; *h*, mtDNA haplotype diversity (± SD). *Fs*, Fu’s FS statistic; *R*_*2*_, Rozas and Ramon-Onsins’ R_2_ statistic; τ, sudden demographic expansion parameter, with 5% and 95% confidence limits; t, true time since population expansion, from τ = 2 μ T (where μ is the substitution rate per gene).

Significantly small values of F_S_ and R_2_ are indicated in bold.

### Specimens collection statement

We declare that all samples analysed in this study were collected complying with institutional, national, and international guidelines.

## Results

### Sequencing results

A COI barcode sequence of 552 bp for *A. joubini* and sequences of various lengths (see Supplementary Dataset File) for seven out of ten British *A. crataegi* were recovered with NGS. These sequences perfectly matched with other *Aporia* barcodes, when using BOLD Identification System (IDS). Further confirmation of their validity was provided by the fact that they grouped with sequences from closely allied taxa. The missing data in NGS sequences (see Supplementary Dataset File) as well as the lack of full-length (658 bp) barcode sequences for three British *A. crataegi* samples can be attributed to amplification failure of the COI gene due to DNA degradation. Despite a match of 97% to the other *Aporia*, one British *A. crataegi* sequence was excluded due to branching far from the other *A. crataegi* sequences (its position in the tree was likely an artifact produced by its short sequence length: 130 bp containing two N's). Sanger sequencing produced full-length DNA barcodes (658 bp) for all *A. crataegi* and one of the four Iranian *A. leucodice* samples, and sequences of various lengths were obtained for the remaining three samples of the Iranian *A. leucodice* (see Supplementary Dataset File).

### Phylogenetic analyses in the subtribe Aporiina

**Figure 1:**
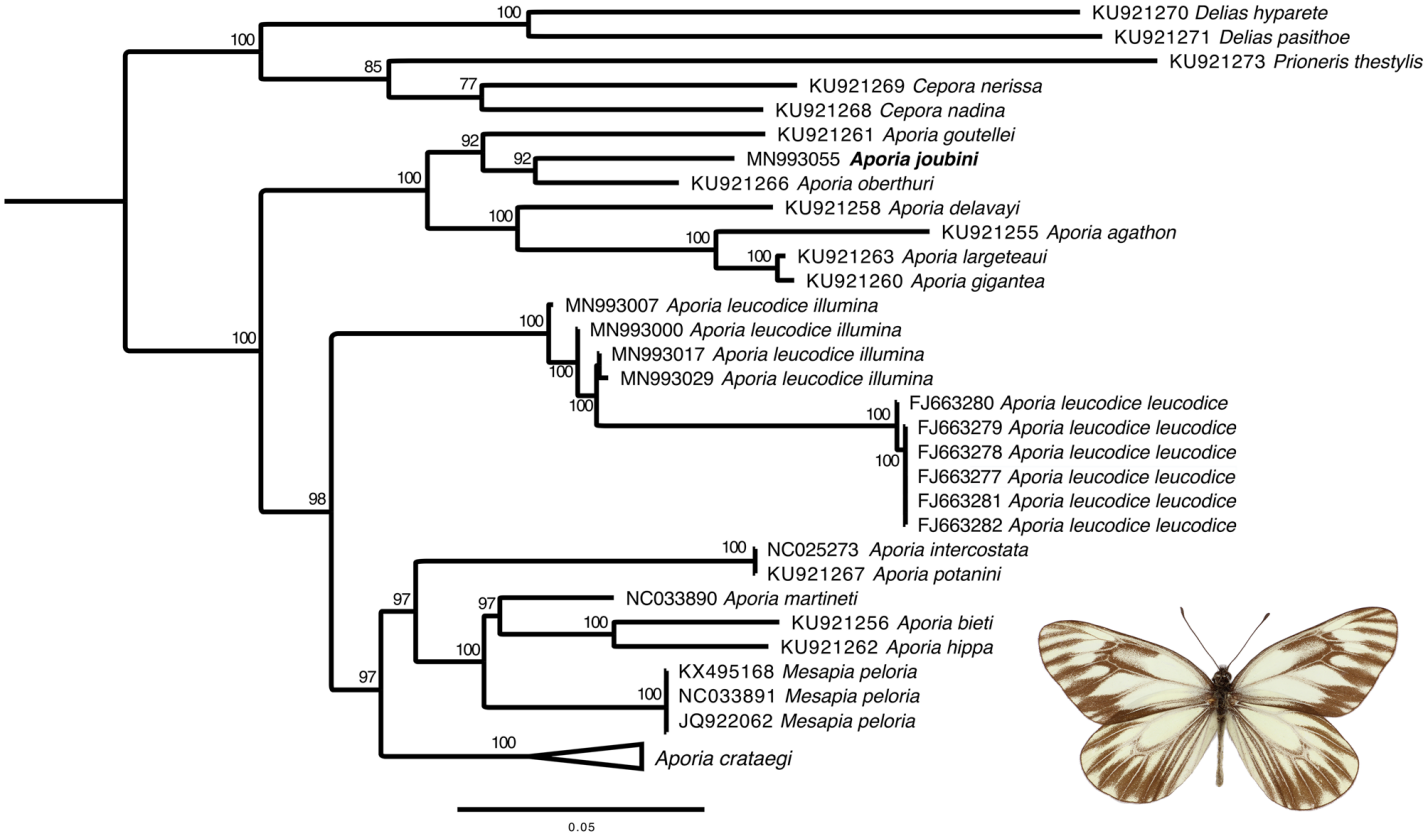
Maximum likelihood (ML) tree of the subtribe Aporiina under GTRCAT model of evolution; numbers above and below branches represent bootstrap support (BS) above 75%. Holotype of *Aporia joubini* NHMUK010201224 (image courtesy of NHM of London: Data Portal https://data.nhm.ac.uk/dataset/56e711e6-c847-4f99-915a6894bb5c5dea/resource/05ff2255-c38a-40c9-b657-4ccb55ab2feb/record/6636649).

The ML tree confirmed the monophyly of *A. crataegi* clade (Bootstrap support, BS = 100) and the close relatedness to a clade (BS = 97) consisting of *Aporia* + *Mesapia* (Fig. 1;^13,18^). Our findings do not corroborate with the morphological groups proposed by Della Bruna et al.^9,19^ based on shape of the uncus in male genitalia. For example, *A. oberthuri* and *A. goutellei* are closely related in our tree (Fig. 1), while the former has a bifid and the latter a pointed apex (see^18^). Our analysis further highlighted that *A. goutellei* (BS = 92) is the sister taxon to a clade consisting of *A. joubini* and *A. oberthuri* (BS = 92). Furthermore, the Iranian populations of *A. leucodice* (ssp. *illumina* Grum-Grishimailo, 1890) appeared distinct, with an average COI barcode distance of 2.67% (± 0.64), from Kazakhstan and Kirghizstan populations (ssp. *leucodice* Eversmann, 1843), forming a highly supported clade (BS = 100).

### Phylogenetic analyses in *Aporia crataegi*

Although our ML results recognized few robust nodes within *A. crataegi* (Supplementary File 1), five main clusters stood out. One (BS = 100) comprised most of the populations from Iran (except IRMEY1 and IRSHI1), Cyprus, and TRERZ1 from East Turkey (Erzurum, Bingöl). A second highly distinctive and strongly supported lineage, including all sequences from Maghreb (Algeria and Morocco; BS = 100), was recovered as the closest relative (BS = 98) to a third distinctive clade with high support (BS = 100) formed by populations from Hokkaido (Japan), Almaty (Kazakhstan) and Yakutia (Russia). A fourth clade included three of the six Sicilian sequences (BS = 92; IBOSC1, ITEOD1, IMART1). Lastly, all mtDNA sequences from the remaining parts of Eurasia (Supplementary File 1) clustered into a large set of closely allied lineages. It is also worth noting that although deep phylogenetic relationships among *A. crataegi* sequences could not be satisfactorily resolved due to very low divergence along internal branches, Eurasian sequences consistently appeared distinct from all other populations in our ML reconstructions.

### Network analysis of the mtDNA haplotypes

**Figure 2:**
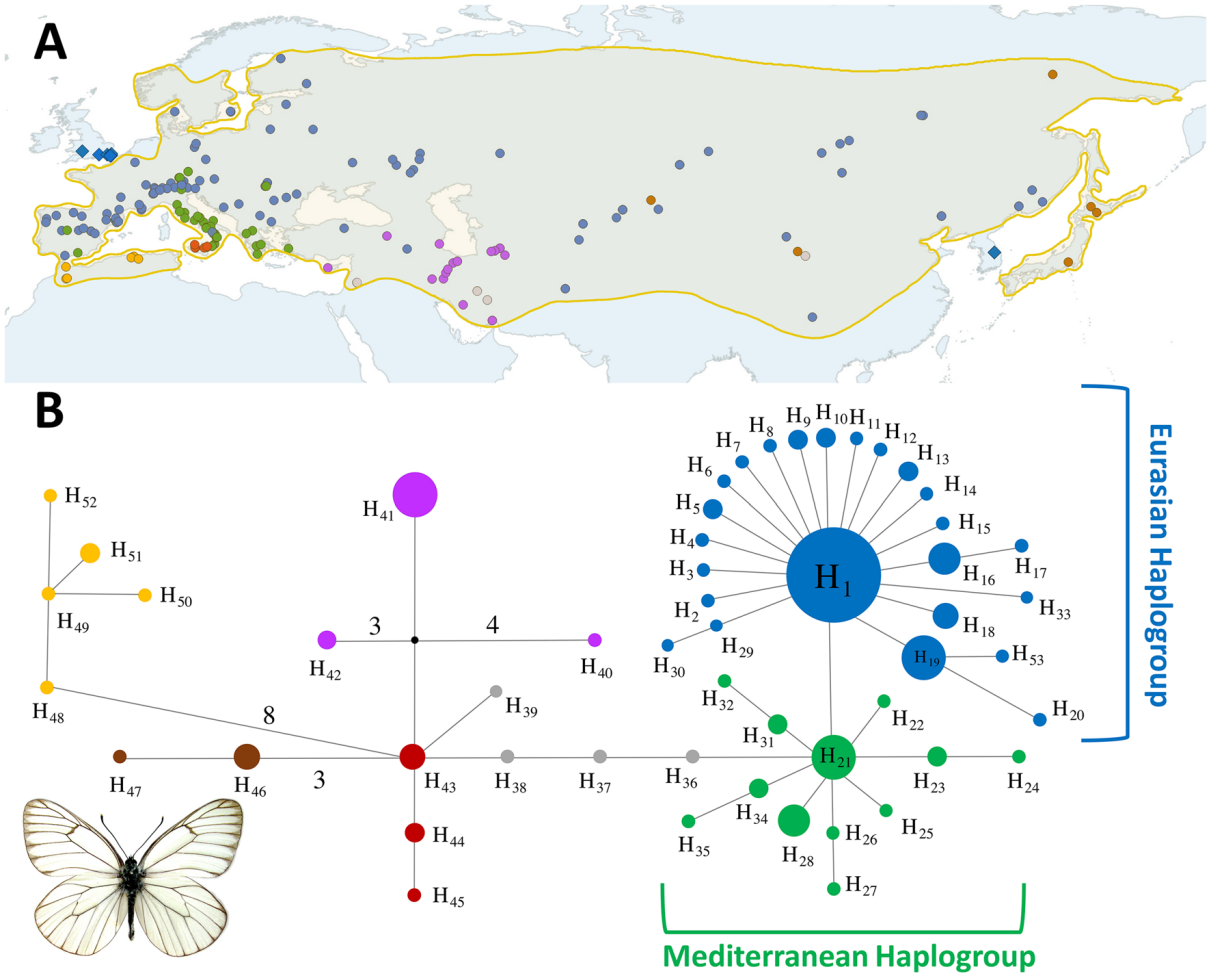
(**A**) Sampling localities and approximate geographic distribution areas of the *Aporia crataegi* redrawn after^9,23,25^. Sampling localities for the populations included in this study are showed with different coloured circles, while the extinct populations are indicated with squares. The map was prepared using Quantum GIS 2.8.2 (https://qgis.org/downloads/) based on a map from Natural Earth (www.naturalearthdata.com). (**B**) Median-Joining Network of *A. crataegi* COI sequences. The size of circles is proportional to haplotype frequency and numbers of mutations between haplotypes are shown at the connections, except for single or double substitutions. In both figures main haplogroups are highlighted and shown in different colours. *Aporia crataegi* image is of a specimen from Uzbekistan (UZTAS: Supplementary Dataset File; image courtesy of Josef de Freina).

In the MJ network analysis, the six sequences of British samples were excluded due to incomplete COI barcode sequences (see Supplementary Dataset File). The 191 analyzed COI barcode sequences showed a total of 53 haplotypes with 45 variable sites (Fig. 2B; Supplementary Dataset File). In this dataset the haplotype (h) and nucleotide diversity (π) was 0.86 (± 0.02) and 0.006 (± 0.011) respectively. Results of this analysis (Fig. 2B) were fully consistent with our ML analysis. Besides populations from Maghreb and Japan that showed several distinct haplotypes well-differentiated from each other, most of the Iranian populations displayed a single haplotype (H_41_) while the Sicilian populations had three haplotypes. The remaining Eurasian sequences, whose phylogenetic relationship were unresolved with ML analysis, were linked with Sicilian populations by three peculiar haplotypes from Iran (H_36_, H_37_) and China (H_38_), and the analysis highlighted two star-like configurations (Fig. 2B). The Eurasian haplogroup (Fig. 2B; haplotype diversity 0.65 ± 0.04, Table 1), included a central haplotype (H_1_) widely distributed from Iberian Peninsula to South Korea, while the Mediterranean haplogroup (haplotype diversity 0.75 ± 0.07, Table 1) consisted mainly of all sequences from Central and South Italy (except for ISIRI1 population from Mt. Sirino, Basilicata), Greece and South Iberian Peninsula.

### Tests of demographic equilibrium and estimation of divergence times

The Fs and R2 statistics, calculated for the Eurasian haplogroup (N = 114) and the Mediterranean haplogroup (N = 35) sequence sets of *A. crataegi* (Fig. 2B), rejected the null hypothesis of constant population size for these two phylogeographic units (Table 1). Mismatch distribution of these groups was examined according to sudden expansion model (Table 1), and goodness of fit tests did not show significant deviations from expected distributions, so that parameter τ = 2μt could be used to estimate the time (t) elapsed from population expansion. Estimated values of τ and their 5% and 95% confidence limits are shown in Table 1. Based on Brower’s^48^ proposed mutation rates in Lepidoptera (µ = 0.01 substitutions/site/lineage/Ma), demographic expansion of the Eurasian and Mediterranean haplogroup could be traced back to about 67 thousand years ago (kya) (49–99 kya) and 116 kya (57–191 kya) respectively.

## Discussion

### The taxonomic status of the genus *Aporia*

The results of our study based on mitochondrial DNA barcode data alone provide strong support for previous findings based on combined data^13,18^ on the systematic relationships within the genus *Aporia* (Fig. 1). The division of *Aporia* into three subgenera (*Aporia*, *Metaporia* and *Mesapia*) is not supported, since both *Aporia* (*Aporia*) and *Aporia* (*Metaporia*) (sensu^19^) are found to be paraphyletic, with *Mesapia* nested within *Aporia *sensu lato. The diagnostic morphological characters for these taxa include smaller wingspan, more rounded wings, and hairy genitalia for *Mesapia*^49^, and minor differences in wing pattern elements for *Metaporia*^9^, none of which, in our opinion, merit a separate subgeneric (sensu^14,15^) or generic (sensu^16,17^) status for these taxa. *Metaporia* has already been synonymized with *Aporia*^9^, and our results highlight a need to also synonymize *Mesapia* under *Aporia* (syn. nov). Within *A. leucodice*, the smaller, darker Iranian populations (ssp. *illumina* Grum-Grishimailo, 1890) appeared considerably distinct from the larger, lighter Kazakhstan and Kirghizstan populations (ssp. *leucodice* Eversmann, 1843). The average COI barcode distance between these two clusters (2.67 ± 0.64%) falls well within the average distance between most *Aporia* species. Despite this, we refrain from elevating *illumina* as a separate species until supporting ecological or morphological evidence become available. As for *A. joubini,* based on similarities in wing pattern, Della Bruna et al.^9^ suspected that this species may represent an aberrant form of *A. harrietae paracraea*, a closely related taxon that flies in the type locality of *A. joubini*. We agree with this assessment. If barcode sequences of *A. hariettae* become available and prove to be very close or identical to *A. joubini*, the latter should be downgraded to a junior synonym of *A. hariettae*.

### Subspecific taxonomy and evolution of *Aporia crataegi*

Within *A. crataegi*, from 23 to 25 subspecies have been described based on fragmented distribution and few morphological characters^9,10^, but our mtDNA analyses reveal a total of six distinct lineages (Fig. 2). Our data highlighted several populations, described as different subspecies, sharing similar or identical mtDNA sequences, suggesting a very recent evolutionary origin. The most evident example occurs in Eurasia, where 66 samples from 53 localities, described as 12 subspecies according to Della Bruna et al.^9^, share an identical haplotype (H1: Fig. 2B) consistent with recent demographic expansion during the Late Pleistocene glacial period (about 67 kya: Table 1) for the Eurasian haplogroup (Fig. 2B). Populations from three major countries in the Mediterranean Basin (Italy, Greece, and Spain: Fig. 2) also showed very weak differentiation, despite three subspecies (ssp. *rotunda*, ssp. *karavaievi*, and ssp. *rutae* respectively) recognized by Della Bruna et al.^9^, confirming a pervasive phylogeographic pattern in European phylogeography^50,51^. The trace of demographic expansion model indeed dates a rapid expansion of the Mediterranean haplogroup to about 116 kya during the Late Pleistocene. The sea-level oscillations during this period, which created land bridges between the three main peninsulas^52^ and permitted faunal exchange, can explain the fragmented distribution of this haplogroup (Fig. 2). This scenario has been suggested as a hypothetical distribution pattern during the last glacial period of many organisms including butterflies (e.g. *Maniola jurtina*^50^). Afterwards, in the post glacial period, gene flow among these populations became interrupted, and they experienced a second expansion towards the continent. It is worth noting that although one mutation separates the two main haplogroups, the present postglacial distribution of the Mediterranean haplogroup in *A. crataegi* (Fig. 2A) appears to be an example of the “grasshopper paradigm”^50^, frequently repeated in many animal and plant species^53,54^ including butterflies (e.g. *Zerynthia polyxena*^55^). The grasshopper paradigm is expected for species showing difficulties in crossing the mountain barriers represented by the Alps and Pyrenees^50,55^. Indeed, in *A. crataegi,* the populations of the Mediterranean haplogroup from Italy and Iberia remained trapped by the Alps and Pyrenees. Conversely, the Greek populations did not expand to Central Europe despite the absence of conspicuous mountain chains that would otherwise impede such colonization. Although the validity of using mtDNA as a marker in molecular ecology has been questioned in the last decade^56,57^, a recent study has provided evidence that mtDNA spatial differentiation is correlated with species traits known to affect the dispersal and colonization capabilities of butterflies^51^. Nevertheless, considering that the mtDNA variation observed in this study is not representative of the whole genomic variation, we cannot exclude a recent expansion of the Greek populations in Central Europe. Nuclear genetic analyses are therefore needed to better investigate the genetic variability, particularly in the contact zones between the main haplogroups. Furthermore, additional sampling might reveal a different genetic pattern in *A. crataegi*.

The extinct British and Korean populations appear to be genetically most closely related to the other specimens from Eurasia (Supplementary File 1). Although this signals possible synonymy of these populations, we refrain from making any changes to their current taxonomy, as any proposal to synonymize a large number of ‘subspecies’ of *A. crataegi* with identical mtDNA haplotypes will require a more in-depth analyses of morphological differences and examination of type materials.

Our samples from five Sicilian populations showed three unique haplotypes, highlighting their significance for biodiversity in Sicily. In Iran, 15 samples from 12 localities encompassing four subspecies (sensu^9^) share an identical haplotype (H41: Fig. 2B). Our analysis strongly supports the distinctiveness of the Iranian populations with the exception of two divergent sequences from Central Iran (Yazd, H36 and Kerman, H37: Supplementary Dataset File). In addition, our results support the distinctiveness of the single sample from Syria (ssp. *augustior*, sensu^9^). As stated above, further sampling and nuclear genetic analyses particularly from Iran, Israel, Syria, Jordan, and Iraq will likely reveal additional haplotypes. Finally, our data supports the genetic peculiarity and evolutionary value of the populations from Japan and north-western Africa, previously recognized as two well-differentiated subspecies (ssp. *adherbal* and ssp. *mauritanica* respectively). Although our phylogenetic analysis doesn’t fully resolve the relationships between the North African and Sicilian populations (ssp. *mauritanica* and ssp. *augusta* respectively), the large differentiation between these populations of *A. crataegi* suggests a prolonged period of lack of genetic exchange between them, likely as a result of an early split in the range of the ancestral stock. Despite the small geographical distance between North Africa and Sicily, eight mutations divide these two subspecies (Fig. 2B). The last land bridge between Europe and Africa is known to have occurred at the end of Miocene (7–5.3 million years ago (mya))^58^, when a temporary closure of the water corridors between Africa and the Iberian Peninsula permitted biotic exchange between the two continents^59^. During this period, known as the ‘Messinian Salinity Crisis’, Spain was connected to Morocco, and Tunisia to Italy via Sicily^60,61^. Re-opening of the Gibraltar at the beginning of the Pliocene (5 mya) restored the barrier. This short period of connection between the two continents has been suggested as a plausible explanation for vicariance between the North African, Iberian, Sicilian and Italian lineages of many organisms, including butterflies (e.g. *Melanargia*^62^; *Pseudophilotes*^63^). However, considering Brower’s^48^ proposed mutation rates for mtDNA in Lepidoptera (µ = 0.01 substitutions/site/lineage/Ma), demographic expansion of major *Aporia crataegi* haplogroups appear to have a much more recent (Late Pleistocene) origin and are possibly influenced by glaciations or other deterministic and stochastic events, as previously noted in other Lepidoptera (e.g. *Polyommatus*^64^; *Aricia*^65^).

## Conclusions

Natural history collections represent an invaluable source of information for species whose ranges are characterized by geomorphological or geopolitical impediments, but principally for storing specimens of extinct taxa when fresh biological material is no longer available, as the case of *A. joubini* and British populations of *A. crataegi*. These repositories allow us to fill the gap in genetic information and reconstruct the evolutionary history of species. For now, NGS-based analysis of COI barcodes has made efficient genomic studies of museum specimens possible, allowing the first look at the genetic traits of extinct species. Rapid advances in modern DNA technology will eventually allow complete reconstruction of genomes, uncovering the lost evolutionary history of these extinct populations and ushering us into a new age of Biological and Conservation Sciences.

## Supplementary information

Supplementary Information

Supplementary Data

## Data Availability

The datasets generated during the current study are available in the BOLD system repository: Dataset name: DS—APORIA; https://doi.org/10.5883/DS-APORIA.
