# Supplementary material for: Next generation sequencing-aided comprehensive geographic coverage sheds light on the status of rare and extinct populations of Aporia butterflies (Lepidoptera: Pieridae)
Source: Sci Rep. 2020 Aug 18;10:13970. doi: 10.1038/s41598-020-70957-4 (PMC7434888; doi:10.1038/s41598-020-70957-4)
Supplement: Supplementary file 1 — Supplementary Information [file 41598_2020_70957_MOESM1_ESM.pdf]

Supplementary_File_1.pdf. Maximum-likelihood (ML) tree of *A. crataegi.* Maximum likelihood (ML) tree of *A. crataegi* populations considered in this study under GTRCAT model of evolution; numbers above and below branches represent bootstrap support (BS) above 75%. The Roman numerals refer to the main groups identified in the ML analysis, and different colours represent main haplogroups (cf. Fig. 2). In brackets the collection date of the extinct population.

Title: Next Generation Sequencing-aided comprehensive geographic coverage sheds light on the status of rare and extinct populations of *Aporia* butterflies (Lepidoptera: Pieridae).

Author List: Valentina Todisco, Raluca Vodă, Sean W. J. Prosser, Vazrick Nazari.

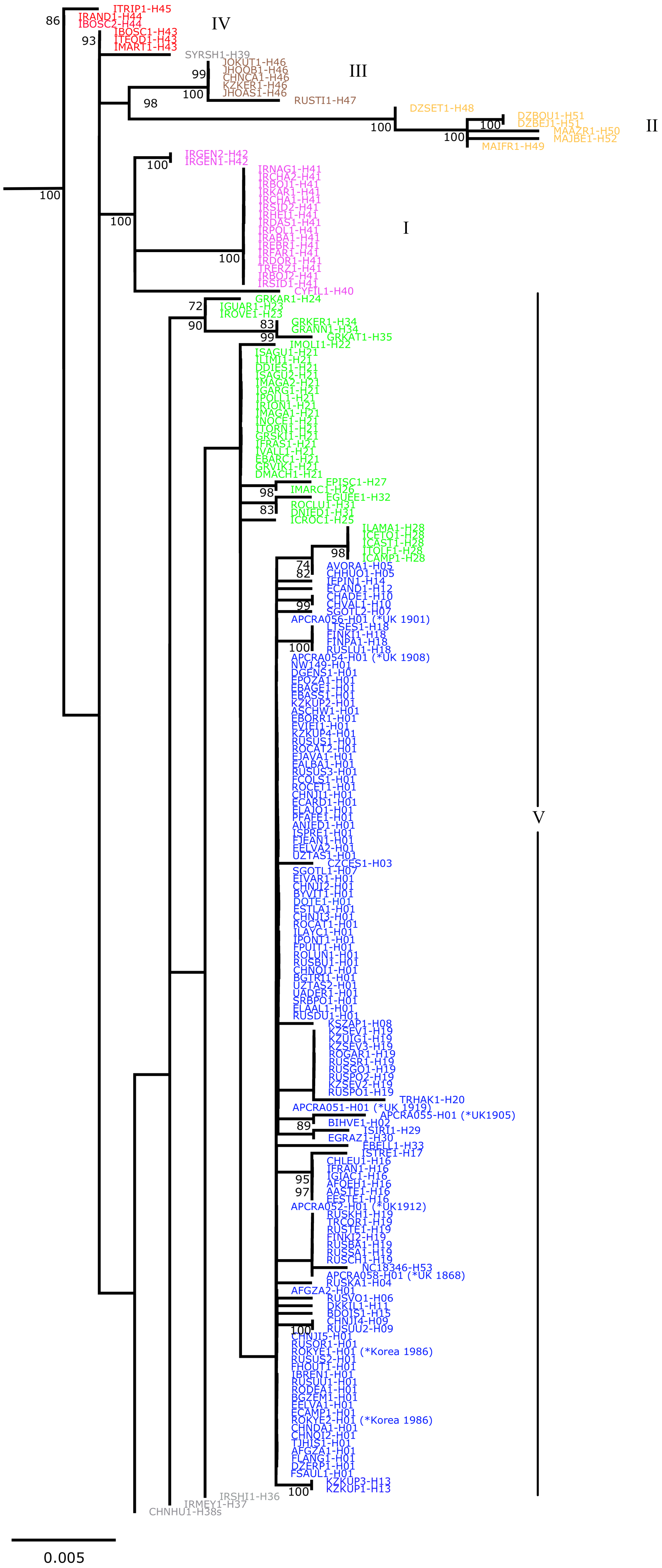

ITRIP1-H45
IRAND1-H44
IBOSC2-H44
IBOSC1-H43
ITEOD1-H43
IMART1-H43
IV
SYRSH1-H39
JOKUT1-H46
JHOOB1-H46
CHNCA1-H46
KZKER1-H46
JHOAS1-H46
III
RUSTI1-H47
DZSET1-H48
DZBOU1-H51
DZBEJ1-H51
MAAZR1-H50
MAJBE1-H52
MAIFR1-H49
II
IRGEN2-H42
IRGEN1-H42
IRNAG1-H41
IRCHA2-H41
IRBOJ1-H41
IRKAR1-H41
IRCHA1-H41
IRSID2-H41
IRSHE1-H41
IRDAS1-H41
IRPOL1-H41
IRABA1-H41
IREBR1-H41
IRFAR1-H41
IRDOR1-H41
TRERZ1-H41
IRBOJ2-H41
IRSID1-H41
I
CYFIL1-H40
GRKAR1-H24
IGUAR1-H23
IROVE1-H23
GRKER1-H34
GRANN1-H34
GRKAT1-H35
IMOLI1-H22
ISAGU1-H21
ILIMI1-H21
DDIES1-H21
ISAGU2-H21
IMAGA2-H21
IGARG1-H21
IPOLI1-H21
IRION1-H21
IMAGA1-H21
INOCE1-H21
ITORN1-H21
GRSKI1-H21
IFRAS1-H21
IVALL1-H21
EBARC1-H21
GRVIK1-H21
DMACH1-H21
EPISC1-H27
IMARC1-H26
EGUEE1-H32
ROCLU1-H31
DNIED1-H31
ICROC1-H25
ILAMA1-H28
ICETO1-H28
ICAST1-H28
ITOLF1-H28
ICAMP1-H28
AVORA1-H05
CHHUO1-H05
IEPIN1-H14
ECAND1-H12
CHADE1-H10
CHVAL1-H10
SGOTL2-H07
APCRA056-H01 (*UK 1901)
TSES1-H18
FINKI1-H18
FINPA1-H18
RUSLU1-H18
APCRA054-H01 (*UK 1908)
NW149-H01
DGENS1-H01
EPOZA1-H01
EBAGE1-H01
EBASS1-H01
KZKUP2-H01
ASCHW1-H01
EBORR1-H01
EVIE1-H01
KZKUP4-H01
RUSUS1-H01
ROCAT2-H01
EJAVA1-H01
EALBA1-H01
RUSUS3-H01
ECOLS1-H01
ROCET1-H01
CHNJI1-H01
ECARD1-H01
ELAJO1-H01
PFAFE1-H01
ANIED1-H01
ISPRE1-H01
EJEAN1-H01
EELVA2-H01
UZTAS1-H01
CZCES1-H03
SGOTL1-H07
EIVAR1-H01
CHNJI2-H01
BYVIT1-H01
DOTE1-H01
ESTLA1-H01
CHNJI3-H01
ROCAT1-H01
ILAYC1-H01
IPONT1-H01
FPUTT1-H01
ROLUN1-H01
RUSBU1-H01
CHNQI1-H01
BGTRI1-H01
UZTAS2-H01
UADER1-H01
SRBPO1-H01
ELAAL1-H01
RUSDU1-H01
KZZAP1-H08
KZSEV1-H19
KZUIG1-H19
KZSEV3-H19
ROGAR1-H19
RUSSR1-H19
RUSGO1-H19
RUSPO2-H19
KZSEV2-H19
RUSPO1-H19
TRHAK1-H20
APCRA051-H01 (*UK 1919)
APCRA055-H01 (*UK1905)
BIHVE1-H02
ISIRI1-H29
EGRAZ1-H30
EBELL1-H33
ISTRE1-H17
CHLEU1-H16
IFRAN1-H16
IGIAC1-H16
AFQEH1-H16
AASTE1-H16
EESTE1-H16
APCRA052-H01 (*UK1912)
RUSKH1-H19
TRCOR1-H19
RUSTE1-H19
FINKI2-H19
RUSBA1-H19
RUSSA1-H19
RUSCH1-H19
NC18346-H53
APCRA058-H01 (*UK 1868)
RUSKA1-H04
AFGZA2-H01
RUSVO1-H06
DKKIL1-H11
BDOIS1-H15
CHNJI4-H09
RUSUU2-H09
CHNJI5-H01
RUSOR1-H01
ROKYE1-H01 (*Korea 1986)
RUSUS2-H01
FHOUT1-H01
IBREN1-H01
RUSUU1-H01
RODEA1-H01
BGZEM1-H01
EELVA1-H01
ECAMP1-H01
ROKYE2-H01 (*Korea 1986)
CHNDA1-H01
CHNQI2-H01
TJHIS1-H01
AFGZA1-H01
ELANG1-H01
DZERP1-H01
FSAUL1-H01
KZKUP3-H13
KZKUP1-H13
V
IRSHI1-H36
IRMEY1-H37
CHNHU1-H38s
0.005
